# The Reciprocal Effects of Prosociality, Peer Support and Psychological Well-Being in Adolescence: A Four-Wave Longitudinal Study

**DOI:** 10.3390/ijerph21121630

**Published:** 2024-12-07

**Authors:** Gaetana Affuso, Nicola Picone, Grazia De Angelis, Mirella Dragone, Concetta Esposito, Maddalena Pannone, Anna Zannone, Dario Bacchini

**Affiliations:** 1 Department of Psychology, University of Campania “Luigi Vanvitelli”, 81100 Caserta, Italy; nicola.picone@unicampania.it (N.P.); magdipan@hotmail.it (M.P.); 2 Department of Psychology and Educational Sciences, Pegaso University, 80143 Napoli, Italy; grazia.deangelis@unipegaso.it; 3 Faculty of Law, Giustino Fortunato University, 82100 Benevento, Italy; m.dragone@unifortunato.eu; 4 Department of Humanistic Studies, University of Naples “Federico II”, 80133 Napoli, Italy; concetta.esposito3@unina.it (C.E.); zannoneanna@gmail.com (A.Z.); dario.bacchini@unina.it (D.B.)

**Keywords:** prosociality, peer support, psychological well-being, cross-lagged panel model, random intercept cross-lagged panel model

## Abstract

The aim of this study was to analyze the reciprocal effects between prosociality, peer support and psychological well-being using a four-wave longitudinal study and a within-person analytical approach (random intercept cross-lagged panel model, RI-CLPM). A sample of 587 adolescents (males = 308; *M*_age_ = 14.23, *SD* = 0.58) enrolled in the first year of high school (9th grade) were recruited and followed over four years from 2016 (Time 1 [T1]) to 2019 (Time 4 [T4]). Once a year, they filled in a questionnaire measuring prosociality, peer support, and psychological well-being. The results from the RI-CLPM revealed that, at the between-person level, prosociality, peer support and psychological well-being were all positively associated. Conversely, at the within-person level and at all survey points, only psychological well-being positively predicted prosociality one year later. Specifically, we found that adolescents with higher levels of psychological well-being were more likely to show a tendency for prosocial behaviors over time. Promoting interventions aimed at enhancing psychological well-being may make adolescents more likely to engage in positive behaviors, such as prosocial ones, in a variety of contexts, thereby creating favorable social environments.

## 1. Introduction

Regardless of their underlying reasons, individuals act in a prosocial way whenever their behaviors are meant to benefit others [1,2]. Such acts (e.g., donating money, volunteering, etc.) can have positive effects for both the helper and the receiver, such as increased well-being and decreased levels of internalizing and externalizing issues among adolescents [3,4,5,6]. Prosociality can also enhance peer relationships [7], which may in turn make individuals engage more in prosocial behaviors through the satisfaction of relatedness needs [8]. Overall, peer relationships represent another valuable resource for adolescents’ well-being since peers and friends become increasingly influential to the detriment of family members throughout this period of development [9,10]. For instance, acceptance from peers was found to protect adolescents against depressive symptoms [11]. Lastly, psychological well-being itself may bring about positive effects on prosociality and peer relationships as evidenced by studies conducted in educational contexts [8,12].

Based upon the results of previous research, the objective of the present study is to analyze the reciprocal influences between prosociality, peer support and psychological well-being in a sample of adolescents followed over four years. The results would inform future interventions aimed to increase adolescents’ psychological well-being consistently with goal three of the 2030 Agenda [13] concerning the promotion of well-being and mental health.

To the best of our knowledge, very few studies have examined the relationships between prosociality, peer support and psychological well-being and their potential mediating roles. Specifically, no study has examined these relationships in high school students and over a four-year time interval. Moreover, given that we were mainly interested in observing how within-person variations in prosociality, peer support and psychological well-being scores are correlated or can be predicted, a random intercept cross-lagged panel model (RI-CLPM; [14]) appeared to be the most appropriate analytical model. RI-CLPMs, which include random intercepts to account for stable unobserved individual differences, separate within-person (time-varying) effects from between-person (time-invariant) effects, providing a clearer understanding of how variables influence each other over time while controlling for individual differences at baseline. To date, no study has examined the reciprocal influences between prosociality, peer support and psychological well-being over four waves using a RI-CLPM, which is recommended when the research question concerns the prospective effects of within-person deviations from trait levels [15].

### 1.1. Prosociality and Psychological Well-Being

Prosociality refers to the individual tendency to help and benefit others [2,16]. Such a concept encompasses a range of behaviors (e.g., helping others in a material way, psychologically, etc.) which are not necessarily related to the moral values of the individual who performs them [1]. That is, a variety of reasons (e.g., gaining status and recognition, among others) may make people engage in a prosocial way; nevertheless, the behavior can be defined as altruistic whenever the driving force is moral [1]. Further, as argued by Luengo Kanacri et al. [17], engagement in prosocial behaviors depends on several factors, including biological, social cognitive and temperamental characteristics as well as socialization and specific situations.

Research on prosociality is part of that current called positive psychology [18], which highlights the beneficial role of certain dimensions (e.g., hope, optimism, etc.) in people’s lives as opposed to a pathological focus on the human being. In line with that, previous studies have investigated the relation between prosociality and health outcomes by using diverse research designs. For instance, in their longitudinal experiment, Nelson et al. [19] showed that participants who were asked to carry out kind behaviors reported higher levels of psychological flourishing compared with participants who were asked to carry out self-focused behaviors. Similarly, in a cross-sectional study involving people from eight regions in the world, Haller et al. [20] revealed that prosociality boosted well-being during the Coronavirus pandemic. Furthermore, some studies indicated that, over time, prosociality was negatively associated with both externalizing (e.g., substance use) and internalizing (e.g., depression) difficulties among adolescents [3,4]. Therefore, fostering prosociality may be useful not only for people receiving them, but also for helpers, consistently with a recent review highlighting the positive impact of prosocial interventions on well-being [21].

The inverse relation may also exist. Individuals with higher levels of psychological well-being are more prone to help others because they may want to keep such psychological states through prosocial acts or they may have more resources to employ in positive behaviors [12,22]. In this regard, some scholars stressed the positive influence of well-being on prosociality [23]. For example, Kushlev et al. [24] showed that some dimensions of well-being (life satisfaction and positive affect) increased prosociality (e.g., donating money and volunteering). Additionally, Liu and Wang [25], by applying a within-person analytical approach (RI-CLPM), found that within-person changes in prosociality and in emotional problems were reciprocally related over time in a sample of adolescents. Consequently, there could be a positive feedback loop in which prosociality and psychological well-being recursively reinforce each other [22].

### 1.2. Prosociality, Peer Support and Psychological Well-Being

Along with prosociality, peer support and acceptance may represent another noticeable source for adolescents’ well-being as revealed by findings about the relation of social support with a range of outcomes such as happiness, self-esteem, and life satisfaction [26,27,28]. Further, social support also has a protective role against mental health issues (e.g., depression and anxiety [29]).

The relationship between prosociality, peer support and psychological well-being may be explained by the Self-Determination Theory (SDT [30]). According to this framework, the fulfillment of autonomy, competence and belongingness needs has a positive impact on people’s wellness (e.g., students’ level of academic success and psychological adjustment). Weinstein and Ryan [6] suggest that prosocial behavior may serve as a pathway to the three needs identified in the SDT. In other words, they argue that voluntary prosocial behaviors may enhance one’s sense of autonomy, but also belongingness and competence, since they let the helper build a closer connection with others (receivers of the help) and feel capable of achieving goals (by helping the receivers). Volition is implied in the concept of prosociality itself. As a matter of fact, Caprara et al. [16] refer to prosociality as people’s willingness to act in a prosocial way. Thus, tending to show prosocial behaviors may improve the helper’s psychological well-being through the fulfillment of the needs identified by the SDT. As further proof of that, Weinstein and Ryan [6] revealed that prosocial behaviors based upon autonomous motives (not upon external instrumental purposes such as gaining higher status) were associated with greater well-being among helpers thanks to their feelings of autonomy, competence, and belongingness. Consequently, peer support may work as a mediator in the relationship between prosociality and well-being; that is, prosociality may enhance positive relationships among students through more peer support and acceptance which may in turn increase psychological well-being. In terms of research evidence, prosociality has been found to have a positive impact on peer acceptance in several types of studies, including cross-sectional and transactional studies using a between-person analytical approach (traditional cross-lagged panel model, CLPM [31,32]). Furthermore, by using a longitudinal experiment, Layous et al. [7] showed that exhibiting kind acts led to higher levels of both peer acceptance and well-being among students, whereas Guo et al. [33] found an indirect impact of prosociality (self and peer-rated) on academic success via peer acceptance.

However, the inverse association may also be true. Including the three variables simultaneously and using a between-person analytical approach (CLPM), Su et al. [8] revealed that prosociality, satisfaction of relatedness needs, and well-being in school positively influenced each other reciprocally over time in a sample of elementary school students. They also pointed out that satisfaction of relatedness needs had a mediating role in the indirect influence of psychological well-being on prosociality and vice versa. Lastly, Xiong et al. [34], using a within-person analytical approach (RI-CLPM) in a longitudinal study involving adolescents, found a positive association between prosociality on the one hand and peer acceptance and well-being on the other hand at the between-person level. Whereas, at the within-person level, they found just a positive bidirectional association between prosociality and peer acceptance.

Overall, results may differ according to the data analysis technique that is used, that would make it complicated to draw conclusions. However, whenever the aim is to analyze how within-person variations in one variable are related to within-person variations in another one over time, a RI-CLPM would be a more appropriate analysis to perform.

### 1.3. The Current Study

Given the mixed results of previous research, the relationships between prosociality, peer support and psychological well-being remain unclear in the literature. To clarify the nature of these relationships, the present study aims to examine the reciprocal effects of prosociality, peer support and psychological well-being in adolescence using a four-wave longitudinal design and a within-person analytical approach (RI-CLPM).

Specifically, we hypothesize that: (a) prosociality would be positively related to later within-person changes in peer support and vice versa; (b) prosociality would be positively related to later within-person changes in psychological well-being and vice versa; (c) peer support would be positively related to later within-person change in psychological well-being and vice versa; and (d) peer support would play a mediating role in the indirect influence of prosociality on psychological well-being and vice versa.

In addition, in view of the differences found between males and females in prosociality, peer support and psychological well-being [35,36,37], the role of gender was included as a covariate influencing all variables.

## 2. Materials and Methods

### 2.1. Participants

The participants were part of the Arzano Longitudinal Study (ALP), a research project that aims to investigate the determinants and pathways of typical and atypical development from early to late adolescence. In 2016, a cohort of students enrolled in the first year of high school (9th grade) was recruited and followed until the fourth year of high school (12th grade). A total of 587 adolescents—308 males (52.5%) and 279 females (47.5%), *M*_age_ = 14.23 years (*SD* = 0.58)—participated in the study. The questionnaires about prosociality, peer support and psychological well-being were administered once a year for four years, from 2016 = T1 to 2019 = T4.

To guarantee the generalizability of the data, students were recruited from several high schools with different specializations (scientific, humanistic, linguistic and technical-professional) in Arzano and Casoria (two districts in the city of Naples, southern Italy) and in the Naples metropolitan area.

### 2.2. Procedure

Written consent was obtained from the headteachers of the participating schools, the adolescents and their parents before the questionnaires were administered. All questionnaires were administered in the classroom during school hours in February and in the presence of specially trained master’s and doctoral students.

The study was approved by the Research Ethics Committee of Psychological Research of the Department of Humanities Studies, University of Naples “Federico II”. The American Psychological Association’s ethical standards for research involving human subjects were followed in the design and conduct of the study.

### 2.3. Measures

#### 2.3.1. Gender

Gender was self-reported and coded as 1 for boys and 2 for girls.

#### 2.3.2. Prosociality

A shortened version of the Prosociality Scale developed by Caprara et al. [16] was used to measure prosociality. It comprises 10 items aimed to assess individuals’ tendency to behave in a prosocial way such as helping and caring for others (e.g., “I try to console others”). Participants reported on the frequency of such acts using a 5-point Likert-type scale from 1 (“Never/Almost never”) to 5 (“Almost always/Always”) such that higher scores represented higher levels of prosociality. Through the four waves, McDonald’s Omegas ranged from 0.90 to 0.92.

#### 2.3.3. Peer Support

The Classroom Life Scale [38,39], in its Italian version [40], was used to measure perceived support that students received from their peers. Only the five item-Personal Support dimension was employed (e.g., “My classmates really care for me”) in which participants were asked to answer on a 5-point Likert-type scale from 1 (“Never”) to 5 (“Always”) such that higher scores represented higher levels of perceived support. In the current study, McDonald’s Omegas ranged from 0.87 to 0.90.

#### 2.3.4. Psychological Well-Being

The Psychological Well-being Scale [41], in its Italian version [42,43], was used to measure psychological well-being. It comprises 18 items (e.g., “Overall I am able to deal with my duties”) that participants were asked to answer using a 6-point Likert-type scale from 1 (“Not my case”) to 6 (“I totally agree”). Some items were reversed in such a manner that higher scores represented higher levels of psychological well-being. In the present study, McDonald’s Omegas ranged from 0.80 to 0.82.

### 2.4. Analytic Strategy

We tested the longitudinal measurement invariance through confirmatory factor analyses (CFAs). Separate CFAs were run for prosociality, peer support and psychological well-being dimensions. The configural, metric and scalar levels of invariance were assessed.

Multivariate Analyses of Variance (MANOVA) (IBM SPSS Statistics 21, Armonk, NY, USA) were used to compare the mean scores of prosociality, peer support and psychological well-being measured at T1 between students who dropped out of the study and those who remained until T4. Correlation coefficients were used to analyze the relationships between the variables. These analyses were carried out using the SPSS package (IBM SPSS Statistics 21, Armonk, NY, USA).

Given that different results may be obtained depending on the analytical approach used, two types of analyses were carried out to test our hypothesis: a traditional cross-lagged panel model (CLPM) and a randomintercept cross-lagged panel model (RI-CLPM; [14]). CLPM, aggregating the between- and within-person sources of variance, assumes that all unobserved heterogeneity (individual differences) is captured by the observed variables and their interactions over time. RI-CLPM, including random intercepts to account for stable unobserved individual differences, separates within-person (time-varying) and between-person (time-invariant) effects, providing a clearer understanding of how variables influence each other over time while controlling for individual baseline differences. Specifically, first a CLPM (only for comparison reasons) with bidirectional paths between prosociality, peer support and psychological well-being was estimated. Moreover, to maximize measurement equivalence of the constructs across waves, we tested whether the stability paths, cross-lagged effects and within-time correlations were time invariant and could therefore be constrained over time. Second, a RI-CLPM was used to examine whether adolescents’ prosociality would be related to later within-person changes in psychological well-being and vice versa, whether adolescents’ prosociality would be related to later within-person changes in peer support and vice versa and whether adolescents’ peer support would be related to later within-person changes in psychological well-being and vice versa. Also, in this case equality constraints across time were included.

The analyses were run in Mplus 7.4 using maximum log-likelihood estimations [44]. The indirect effects were calculated using the indirect effect test implemented in Mplus 7.4. We used several indexes to determine model fit: the comparative fit index (CFI [45]), the Tucker–Lewis index (TLI [46]), and the root mean square error of approximation (RMSEA [47]). A CFI and TLI ≥ 0.90 and an RMSEA ≤ 0.08 indicate a model’s acceptable fit to the data [48]. The Satorra–Bentler chi-square difference test (ΔSBχ^2^) was used to compare the fit of nested models [49].

## 3. Results

### 3.1. Longitudinal Measurement Invariance

The results regarding longitudinal invariance testing for prosociality, peer support and psychological well-being are reported in Table 1. Partial scalar invariance was achieved for all measures by freeing one or more intercepts, allowing us to compare factor means [50]. Partial invariance is a compromised way to handle the lack of invariance, relaxing noninvariant items while other invariant items are still constrained [50].

### 3.2. Attrition Analysis and Descriptive Statistics

Attrition analysis revealed that 154 (26.2%) adolescents (94 males, 30.5%; 60 females, 21.5%) of the original 587 participants dropped out of the study over time. MANOVA revealed significant differences between those who dropped out and those who remained until T4 (Wilks’s λ = 0.97, *F* (3,562) = 4.98, *p* = 0.002). Participants who dropped out of the study had lower scores for prosociality and psychological well-being at T1. The results of the Little test [51] for missing data completely at random (MCAR) in SPSS version 21 were significant (χ^2^ = 337.301, df = 277; *p* = 0.008), indicating that the data were not missing completely at random. Accordingly, full information maximum likelihood (FIML) was used in CLPM and RI-CLPM for the treatment of missing data. FIML does not estimate missing data, but instead directly fits the model of the covariance structure to the observed raw data.

Means, SDs and correlations of the study variables can be found in Table 2. Prosociality, peer support and psychological well-being were positively associated at each time point. Female gender was significantly and positively associated with prosociality and with psychological well-being at T4 and T5.

### 3.3. Traditional Cross-Lagged Panel Model

First, a traditional cross-lagged panel model was estimated to examine the bidirectional relationships between prosociality, peer support and psychological well-being. The fit for this model was χ^2^(27) = 213.15, *p* < 0.001, RMSEA = 0.10, TLI = 0.76, CFI = 0.92. Then, the paths across time were constrained to be equal and the fit of this model was χ^2^(63) = 354.82, *p* < 0.001, RMSEA = 0.08, TLI = 0.84, CFI = 0.87. The delta chi-square statistic showed that the fit of the constrained model across time was significantly worse than that of the unconstrained model (Δχ^2^(36) = 141.68, *p* < 0.001). Examination of the modification indices suggested that we could improve the fit of this model by estimating the path from peer support at T2 to prosociality at T3, the path from gender to prosociality at T1 and to peer support at T1, the within-time correlations at T1, and the correlation between prosociality and peer support at T2, freely across time. After this refinement, the model fit the data well (χ^2^(56) = 251,137, *p* < 0.001, RMSEA = 0.07, TLI = 0.88, CFI = 0.91). Therefore, we chose this last model with partial invariance across time because it showed better fit and a non-significant delta chi-square statistic (Δχ^2^(29) = 37.99, *p* = 0.12).

**Figure 1:**
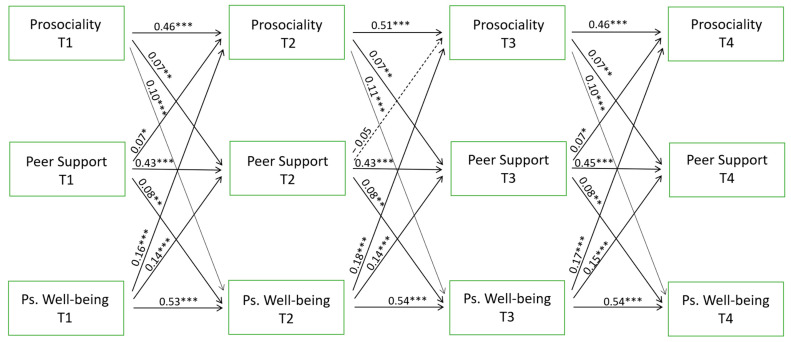
Standardized estimates from the CLPM. Solid and dotted lines represent significant and non-significant paths, respectively. * *p* < 0.05 ** *p* < 0.01 *** *p* < 0.001. To simplify, the paths from gender as a covariate to all variables, and within-time correlations are omitted.

The results (Figure 1) showed that all autoregressive paths and all reciprocal effects were positive and significant, except that between peer support at T2 and prosociality at T3. All within-time correlations were positive and significant at *p* < 0.01. Female gender (1 = boys, 2 = girls) was significantly and positively associated with psychological well-being and prosociality at all survey points, and significantly and positively associated with peer support only at T1 (Table 3). Therefore, being a girl was associated with increased levels of psychological well-being and prosociality at all survey points and with increased levels of peer support at T1. Indirect effects analysis showed that prosociality at T1 and T2 significantly and indirectly influenced psychological well-being at T3 and T4 through peer support at T2 and T3 (β = 0.01, *p* < 0.05; β = 0.01, *p* < 0.05) and that psychological well-being at T2 significantly and indirectly influenced prosociality at T4 through peer support at T3 (β = 0.01, *p* < 0.05).

### 3.4. Random Intercept Cross-Lagged Panel Model

A random intercept cross-lagged panel model was then estimated to examine the bidirectional relationships between prosociality, peer support and psychological well-being, separating between- and within-person sources of variance. The fit for this model was χ^2^ (21) = 35.83, *p* = 0.02, RMSEA = 0.03, TLI = 0.97, CFI = 0.99. Subsequently, the paths across time were constrained to be equal, and the fit for this model was χ^2^ (57) = 120.53, *p* < 0.001, RMSEA = 0.04, TLI = 0.96, CFI = 0.97. The delta chi-square statistic showed that the fit of the constrained model across time was significantly worse than that of the unconstrained model (Δχ^2^ (36) = 84.7, *p* < 0.001). The examination of the modification indices suggested that we could improve the fit of this model by estimating the path from gender to psychological well-being at T1 and the correlation between peer support and psychological well-being at T1, freely across time. After this refinement, the model fit the data well (χ^2^ (54) = 79.01, *p* = 0.01, RMSEA = 0.02, TLI = 0.98, CFI = 0.99). Therefore, we chose this last model with partial invariance across time because it showed better fit and a non-significant delta chi-square statistic (Δχ^2^ (33) = 43.18, *p* = 0.11).

At the between-person level, the results revealed that prosociality was positively associated with peer support (β = 0.53, *p* < 0.001) and psychological well-being (β = 0.51, *p* < 0.001) and that peer support was positively associated with psychological well-being (β = 0.72, *p* < 0.001).

**Figure 2:**
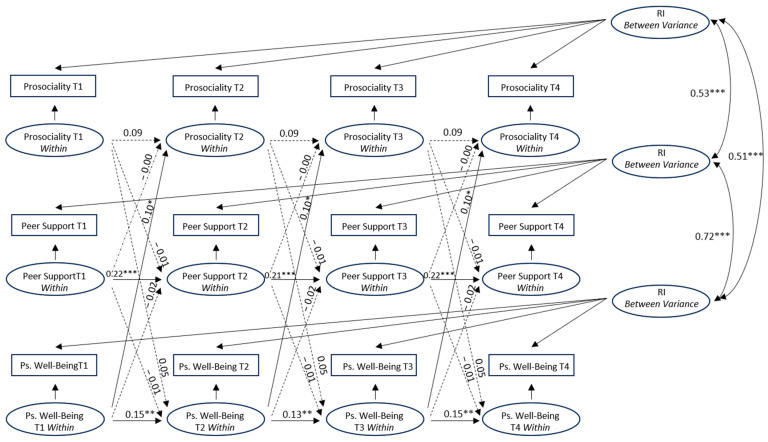
Between-person and within-person standardized estimates from the RI-CLPM. Solid and dotted lines represent significant and non-significant paths, respectively. * *p* < 0.05 ** *p* < 0.01 *** *p* < 0.001. To simplify, the paths from gender as a covariate to all variables, and within-time correlations are omitted.

At the within-person level (Figure 2), only the autoregressive paths of peer support and psychological well-being were statistically significant over time. Adolescents who reported higher than usual levels of peer support and psychological well-being showed an increase in peer support and psychological well-being one year later. The cross-lagged paths suggested that only those adolescents who reported higher than usual levels of psychological well-being showed an increase in prosociality one year later.

The results described so far emerged by controlling the relationships for gender, which was included in the analysis as a covariate. Specifically, female gender (1 = boys, 2 = girls) was positively associated with psychological well-being and prosociality at all survey points (Table 4). Therefore, being a girl was associated with increased levels of psychological well-being and prosociality at all survey points. Within-time correlations between peer support and psychological well-being and between peer support and prosociality were positively and significantly associated at all survey points at *p* < 0.01.

Finally, to understand which model fits the data better, the fit of both models was compared using Akaike’s Information Criterion (AIC). The lower AIC suggests that the RI-CLPM (AIC = 12,551.3) fits the data better than the CLPM (AIC = 12,719.4).

## 4. Discussion

The current study aimed to explore the reciprocal relationships between prosociality, peer support and psychological well-being in a sample of adolescents from high schools followed over four years from 2016 (T1) to 2019 (T4). Given that different results may emerge according to the analytical approach that is used [8,34], we performed two kinds of analyses (CLPM and RI-CLPM) to shed more light on the nature of such relations. Compared with the CLPM, the RI-CLPM allows researchers to disentangle the between-person variance from the within-person one; thus, it gives us the possibility to show whether within-person changes in one variable are related to within-person changes in another variable that was consistent with our primary aim.

In our study, the results from the CLPM that we used just for a comparative purpose, showed that at all time points there were positive reciprocal influences between the three variables except for peer support at T2 with prosociality at T3. They also showed that peer support had a mediating role in the effects of prosociality at T1 and T2 on psychological well-being at T3 and T4. It also mediated the effect of psychological well-being at T2 on prosociality at T4. Such findings are consistent with the longitudinal study conducted by Su et al. [8] who, by applying a CLPM, found that the three variables reciprocally influenced each other in a sample of elementary school students.

Different results emerged from the RI-CLPM in our investigation. At the between-person level, we showed that prosociality, peer support and psychological well-being were all positively associated. On the contrary, at the within-person level, the autoregressive paths showed that peer support and psychological well-being, but not prosociality, had an increase over time. Therefore, after controlling for individual baseline differences, adolescents who reported higher than usual levels of peer support and psychological well-being showed an increase in peer support and psychological well-being, respectively. Moreover, always at the within-person level, we found that only psychological well-being positively predicted an increase in the levels of prosociality one year later. Therefore, adolescents who reported higher than usual levels of psychological well-being were more likely to show a tendency to behave in a prosocial way one year later.

The findings at the between-person level are consistent with previous research [8] and may be due to stable individual traits underlaying the associations between prosociality, peer support and psychological well-being (e.g., the association of the honesty-humility trait with both prosociality and psychological well-being [52,53]).

On the contrary, the findings at the within-person level may be accounted for by some theories and previous studies. Specifically, according to Carlson et al. [54], there are several hypotheses that may explain the positive effect of psychological well-being on prosociality. For instance, following the focus of attention hypothesis [55], individuals in a good mood may perceive themselves as being in a more advantageous state compared with others, which would enhance prosocial behaviors aimed at creating a more equal and fairer social climate. Furthermore, according to the social outlook theory [56], positive social events (e.g., good interactions with others) promote one’s psychological well-being which fosters positive thoughts (e.g., those concerning the goodness in individuals) which in turn drive people to behave in a prosocial way. Carlson et al. [54] also support the mood maintenance hypothesis [22,57], that is, happy individuals may engage more in helping behaviors to the extent that these acts become ways to maintain their psychological condition. That is consistent with the idea of a positive feedback loop in which psychological well-being and prosociality recursively reinforce each other [22,23]. Lastly, according to the broaden-and-build model [22,58], psychological well-being could extend one’s capacity of thinking and acting kind behaviors to make social connections which, from an evolutionary point of view, are crucial to one’s survival. In terms of research findings, Kushlev et al. [24] showed that two dimensions of psychological well-being (life satisfaction and positive affect) positively affected prosociality among participants from different countries. Such association was also confirmed by a study about the role of well-being in donation behaviors [59]. Conversely, Xiong et al. [34], by applying a RI-CLPM and including simultaneously the three variables examined in the current study, found no association between psychological well-being and prosociality over time. However, these authors covered a shorter time interval (six months) than ours and used non-self-report measures (for prosociality and peer acceptance), which may have led to different results. Lastly, it is noteworthy that some factors, including agreeableness, self-transcendence and empathic self-efficacy beliefs, have been found to be associated with prosociality over time through a mediational path such that they would all represent “layers” of personality [60]. Our results about the role of well-being may extend to such a path. Further investigation is needed.

Regarding gender, treated as a covariate in our analyses, we found that it had a positive association with prosociality and psychological well-being at each survey point. As such, being female was associated with higher levels of both variables. The result is in line with previous research showing that girls, compared with boys, are more likely to act prosocial behaviors (particularly, those socially associated with women) [37] and experience positive effects [35]. That may be explained by gender socialization which encourages girls, during their development, to take on social roles and adhere to norms promoting kindness and care for others [61]. Nonetheless, the gender disparity that we found in psychological well-being is not supported by other studies in which women reported worse mental health outcomes than men [62,63]. This result requires further verification.

The current study is not without limitations. First, although we used a transactional design, we must be cautious when interpreting causal relations given that other variables, not included in the current investigation, might have played a certain role. For example, we could have considered the weight of individual variables such as empathy (in line with the empathy-altruism hypothesis) [64] or social ones such as individualist and collectivist orientations [65]. Moreover, although the concept of prosociality as an individual tendency suggests one’s volition to show prosocial behaviors [16] in line with Weinstein and Ryan’s model [6], we could have measured motivational variables as well (e.g., intrinsic motivation) [66]. Second, we only used self-report measures, therefore, future research is needed to include multi-informant instruments.

However, the present study also presents noticeable strengths. To the best of the authors’ knowledge, it is the first investigation examining the reciprocal relationships between prosociality, peer support and psychological well-being among adolescents followed over a considerable amount of time. Furthermore, compared with previous studies [8,34], we used two types of analytical approaches (CPLM and RI-CLPM) through which we were able to compare different results and disentangle the between-person variance from the within-person one clarifying the specific impact that variables have on one another.

## 5. Conclusions and Practical Implications

The comparison between the two models appears to be fruitful, as it allows one to move towards more effective forms of intervention. While the results of the traditional CLPM suggest thinking about multi-level interventions aimed at increasing prosociality and improving the quality of peer relationships and psychological well-being (because everything seems to be linked to everything), the results of the RI-CLPM (which was better adapted to the data than the CLPM) point to more targeted forms of intervention, which is useful in case resources are scarce. In particular, the RI-CLPM results show that adolescents with higher levels of psychological well-being are more likely to exhibit a tendency for prosocial behaviors over time. As argued by the World Health Organization (WHO [67]), adolescents’ well-being can be increased through several actions on both the individual and contextual levels. In this regard, schools may provide curricula which enhance adolescents’ self-esteem, confidence and personal skills (e.g., self-regulation and problem solving) [67]. Moreover, they may strengthen their social, emotional and physical environments (e.g., fostering teachers’ skills or providing safe spaces to prevent bullying) as well as promote health services (e.g., giving information to families about mental health services) [67]. Finally, other useful contextual interventions may include changes in governance systems (e.g., dissemination of school policies) and in community partnerships (e.g., developing connections and groups) [67]. Overall, implementing interventions which are primarily aimed at enhancing psychological well-being may make adolescents more likely to engage in positive behaviors, such as prosocial ones, in a variety of contexts, thereby creating favorable social environments.

## Figures and Tables

**Table 1:** Measurement invariance tests across time-points.

	χ^2^ (df)	∆χ^2^ (∆df)	*p*(∆χ^2^)	CFI	TLI	RMSEA
Psychological well-being						
Configural model	249.254 (134)			0.97	0.97	0.04
Metric model	268.508 (149)	19.254 (15)	0.20	0.97	0.97	0.04
Scalar model	313.709 (161)	45.20 (12)	<0.001	0.97	0.96	0.04
Partial scalar model	282.743 (158)	14.235 (9)	0.11	0.97	0.97	0.04
Prosociality						
Configural model	124.914 (74)			0.99	0.98	0.03
Metric model	144.765 (86)	19.851 (12)	0.7	0.99	0.98	0.03
Scalar model	176.763 (95)	31.998 (9)	<0.001	0.98	0.98	0.04
Partial scalar model	157.214 (94)	12.449 (8)	0.13	0.99	0.98	0.03
Peer support						
Configural model	238.422 (134)			0.98	0.98	0.04
Metric model	254.084 (149)	15.662 (15)	0.40	0.98	0.98	0.04
Scalar model	297.192 (161)	43.108 (12)	<0.001	0.98	0.97	0.04
Partial scalar model	267.248 (158)	13.166 (9)	0.16	0.98	0.98	0.03

**Table 2:** Means, SDs and correlations of the study variables.

	1	2	3	4	5	6	7	8	9	10	11	12	13
1. Gender	-												
2. Prosociality —T1	0.27 **	-											
3. Peer Suport —T1	0.06	0.41 **	-										
4. Psychological Well-being—T1	−0.08	0.31 **	0.44 **	-									
5. Prosociality —T2	0.18 **	0.54 **	0.31 **	0.36 **	-								
6. Peer Support —T2	0.03	0.31 **	0.53 **	0.32 **	0.40 **	-							
7. Psychological Well-being—T2	0.04	0.33 **	0.36 **	0.63 **	0.34 **	0.37 **	-						
8. Prosociality —T3	0.21 **	0.51 **	0.31 **	0.31 **	0.56 **	0.23 **	0.33 **	-					
9. Peer Support —T3	−0.01	0.16 **	0.43 **	0.29 **	0.20 **	0.52 **	0.30 **	0.19 **	-				
10. Psychological Well-being—T3	0.13 **	0.31 **	0.26 **	0.51 **	0.30 **	0.26 **	0.61 **	0.32 **	0.29 **	-			
11. Prosociality —T4	0.22 **	0.49 **	0.25 **	0.29 **	0.48 **	0.25 **	0.31 **	0.55 **	0.25 **	0.31 **	-		
12. Peer support —T4	0.01	0.22 **	0.35 **	0.33 **	0.29 **	0.46 **	0.39 **	0.21 **	0.53 **	0.34 **	0.29 **	-	
13. Psychological Well-being—T4	0.16 **	0.33 **	0.32 **	0.51 **	0.31 **	0.33 **	0.62 **	0.32 **	0.33 **	0.58 **	0.38 **	0.41 **	-
M (SD)	1.52 (0.50)	3.55 (0.78)	3.24 (0.95)	4.35 (0.83)	3.67 (0.78)	3.34 (0.90)	4.33 (0.81)	3.81 (0.75)	3.33 (0.92)	4.37 (0.78)	3.82 (0.79)	3.39 (0.89)	4.43 (0.78)

Note: Gender: 1 males, 2 females. Listwise participants. ** *p* < 0.01.

**Table 3:** Standardized effects of gender on all measures from the CLPM.

	PROS T1	PS T1	PWB T1	PROS T2	PS T2	PWB T2	PROS T3	PS T3	PWB T3	PROS T4	PS T4	PWB T4
Gender	0.27 ***	0.13 **	0.04 *	0.10 ***	−0.03	0.04 *	0.11 ***	−0.03	0.04 *	0.10 ***	−0.03	0.04 *

Note: Gender: 1 males, 2 females. * *p* < 0.05, ** *p* < 0.01, *** *p* < 0.001. PROS = prosociality, PS = peer support, PWB = psychological well-being.

**Table 4:** Standardized effects of gender on all measures from the RI-CLPM.

	PROS T1	PS T1	PWB T1	PROS T2	PS T2	PWB T2	PROS T3	PS T3	PWB T3	PROS T4	PS T4	PWB T4
Gender	0.27 ***	0.04	0.10 *	0.28 ***	0.04	0.10 *	0.29 ***	0.04	0.10 *	0.28 ***	0.04	0.10 *

Note: RI-CLPM, Gender: 1 males, 2 females. * *p* < 0.05, *** *p* < 0.001. PROS = prosociality, PS = peer support, PWB = psychological well-being.

## Data Availability

Data are available from the corresponding author on reasonable request.
